# Molecular Detection and Genetic Characterization of Feline Immunodeficiency Virus (FIV) in Seropositive Cats in Northern Italy

**DOI:** 10.3390/pathogens13060463

**Published:** 2024-05-31

**Authors:** Andrea Balboni, Veronica Facile, Laura Gallina, Maria Chiara Sabetti, Francesco Dondi, Mara Battilani

**Affiliations:** 1 Department of Veterinary Medical Sciences, Alma Mater Studiorum-University of Bologna, Ozzano Emilia, 40064 Bologna, Italy; a.balboni@unibo.it (A.B.); veronica.facile2@unibo.it (V.F.); f.dondi@unibo.it (F.D.); mara.battilani@unibo.it (M.B.); 2 Department of Veterinary Sciences, University of Parma, 43126 Parma, Italy; mariachiara.sabetti@unipr.it

**Keywords:** blood, feline immunodeficiency virus, FIV, Italy, phylogeny, PCR, retrovirus, seropositive cats

## Abstract

Feline immunodeficiency virus (FIV) is responsible for immunodeficiency syndrome in cats. Several viral subtypes have been identified, each with a variable geographical distribution. To date, the subtype B is known to be the genotype spread in Italy. In this study, the genetic diversity of FIV in northern Italy was assessed by detecting proviral DNA in the blood samples of 50 cats determined to be positive through an anti-FIV antibodies test. These cats were tested using six different PCR assays, and the identified viruses were sequenced and analyzed. Forty-eight cats were confirmed positive, and several FIV subtypes were characterized. As expected, the subtype B was the most commonly observed, and the subtype A was reported for the first time in Italy. Moreover, a new taxon possibly representing an additional FIV subtype was detected, and one virus belonging to subtype B potentially had a recombinant origin. The genetic variability between the FIV viruses that emerged in this study may lead to the potential diagnostic failure of single molecular tests. Therefore, a new diagnostic strategy, which adopts different molecular tests and sequencing, is recommended to monitor the evolution and spread of FIV.

## 1. Introduction

Feline immunodeficiency virus (FIV) is an RNA lentivirus belonging to the Retroviridae family, causing lifelong persistent infection, and it is responsible for feline immunodeficiency syndrome in domestic cats. The proviral DNA integrates into the host genome, affecting the functionality of the target lymphocytes; this may lead to progressive immunologic dysregulation [1,2]. The progressive immunopathology predisposes to severe and potentially lethal secondary diseases. Specifically, the most common clinical signs include anorexia, fever, lymphadenopathy, gingivostomatitis, hematological disorders, secondary chronic inflammations and neoplasia [3]. FIV infection is endemic in the domestic cat population worldwide, but several genetically and antigenically distinguishable viral subtypes are recognized and show variable geographical distribution [4]. There are currently seven recognized FIV subtypes based on the diversity of V3–V5 hypervariable regions of the env gene: A, B, C, D, E, F, and U-NZenv [5,6]. Furthermore, several recombinant sequences have been identified and classified, for example, between subtypes A and B, subtypes B and D, and subtypes A and C [7]. To date, few older surveys evaluating the spread of the different FIV subtypes in cats in Italy are available in the literature, and subtype B was largely prevalent [8]. The prevention and control of disease caused by FIV is primarily based on the identification and isolation of infected subjects by point-of-care testing to detect anti-FIV antibodies [9,10]. Nevertheless, in point-of-care serological tests, false-positive and false-negative results can occur [4,9]. Direct tests, such as molecular assays, should also be used for diagnosing FIV infection [9]. In particular, proviral DNA is detectable in the peripheral blood lymphocytes from two weeks post-infection and, as cats remain infected for life, it remains potentially detectable until death [2]. Additionally, for these assays, false negative results are possible due to the low amount of circulating provirus. Furthermore, the extreme genetic variability of FIV reduces the sensitivity of the tests [9]. The study of the molecular epidemiology of this virus in different geographical areas is of utmost importance to establish diagnostic strategies based on the detection of viral nucleic acids influenced by the genetic diversity among local subtypes. These difficulties linked to the high genomic variability of FIV highlighted the need for comprehensive diagnostic approaches, which include both serological and molecular tests. The aim of this study was to evaluate the genetic diversity of FIV in northern Italy by detecting proviral DNA in blood samples from seropositive cats and genetically characterizing the identified viruses.

## 2. Materials and Methods

### 2.1. Inclusion Criteria, Sampling and Groups

All cats that tested positive for the presence of anti-FIV antibodies between 2018 and 2021 at the Veterinary Teaching Hospital (VTH), University of Bologna, were retrospectively retrieved from medical records and included in the study. FIV seropositive cats were excluded if a sufficient amount of stored blood sample (200 µL) was not available to perform molecular testing. Only seropositive cats were included to increase the probability of detecting proviral DNA. Cats were tested for anti-FIV antibodies if they had clinical signs or clinicopathological alterations or risk factors related to infection, or for screening purposes. Blood sampling was performed by venipuncture and, for each cat, plasma, serum, or whole blood samples were tested for the presence of anti-FIV antibodies (anti-p15, anti-p24 and anti-gp40) and FeLV p27 antigen using a commercial point-of-care ELISA based test (SNAP FIV/FeLV ComboPlus, IDEXX, Westbrook, ME, USA. For anti-FIV antibody detection, the manufacturer reported a sensitivity of 93.5% [95% CL: 81.7–98.3%] and a specificity of 100% [97.6–100%], https://www.idexx.co.uk/en-gb/veterinary/support/documents-resources/snap-fiv-felv-combo-test-resources/, accessed 10 April 2024). FIV and FeLV point-of-care tests were carried out within 1 h from the sampling, and surplus blood samples were stored at −20 °C until DNA extraction. Only stored surplus material derived from blood samples collected by clinicians for diagnostic purposes following the owner’s informed consent were used. Signalment data, clinical signs and clinicopathological findings of enrolled cats were retrieved from medical records. The enrolled cats with clinical signs or clinicopathological abnormalities potentially referable to FIV infection [1,4,11] and reported in Table S1 were included in the symptomatic cats (SC) group, and healthy cats or cats with clinical signs or clinicopathological abnormalities not referable to FIV infection were included in the asymptomatic cats (AC) group.

### 2.2. Detection of FIV Proviral DNA

DNA extraction from 200 µL of K3EDTA blood samples was carried out by using the NucleoSpin Tissue Kit (Macherey-Nagel, Düren, Germany). Extracted DNA was stored at −20 °C until use. To detect the FIV provirus, DNA extract of each cat included in the study was tested with the following six PCR assays. The primers used for the PCR assays were selected from the scientific literature, preferring those universally used and that reported high sensitivity and specificity, or de novo designed and modified based on a nucleotide FIV sequence alignment. For this purpose, eight complete genome reference sequences of FIV available in the GenBank database (https://www.ncbi.nlm.nih.gov/genbank, accessed 1 December 2020) were selected as representative of the most important and widespread subtypes based on the data available in the literature. The nucleotide alignment was constructed using the ClustalW method implemented in the BioEdit sequence alignment editor version 7.2.5 software (open access software). The reference FIV sequences used for nucleotide alignment are reported in Table S2.

A PCR assay amplifying the FIV highly conserved long terminal repeats (LTR) region was developed in this study for screening purposes. Primers were designed in nucleotide sequence regions conserved among different FIV subtypes, and degenerate bases were added when the alignment showed different residues at the same nucleotide position. Five PCR assays, named from A to E, amplifying fragments of different length of the FIV highly variable env gene, were used to identify circulating FIV subtypes sequencing the amplified products. For this purpose, the primers published and validated by Kann and colleagues [12] were used; two of them were partially modified to increase their specificity toward subtype B (the viral variant known to be circulating in Italy), as they showed mismatches with the reference sequences of this subtype. PCRs A and B targeted the V3–V4 hypervariable regions, PCRs C and D targeted the V4–V5 hypervariable regions, and PCR E targeted the V3–V5 hypervariable regions. All the PCR assays, primer nucleotide sequences, genome positions and amplicon fragment sizes are reported in Table 1. The LTR reaction was performed using the Taq DNA Polymerase Kit (Qiagen, Hilden, Germany), and thermal cycling consisted of an initial denaturation at 95 °C for 5 min, followed by 40 cycles of denaturation at 95 °C for 30 s, annealing at 60 °C for 30 s, and elongation at 72 °C for 30 s, followed by a final elongation step at 72 °C for 7 min. The env PCR assays from A to E were carried out with a proofreading DNA polymerase (Phusion Hot Start II High-Fidelity DNA Polymerase, Thermo Fisher Scientific, Waltham, MA, USA), and thermal cycling consisted of an initial denaturation at 98 °C for 30 s, followed by 40 cycles of denaturation at 98 °C for 10 s, annealing at different temperatures depending on the primers used for 20 s, and elongation at 72 °C for 40 s, followed by a final elongation step at 72 °C for 7 min. Given the different nucleotide sequences of the primers used, the annealing temperatures of the end-point PCRs from A to E were evaluated by performing gradient PCRs to ensure the best assay performance and set at 57, 56, 58, 59 and 59 °C, respectively. For each reaction, a DNA extract of FIV positive sample was used as positive control, and a no template control, consisting of ultrapure water, underwent analysis simultaneously.

### 2.3. Sequence Analysis

The PCR products obtained from amplification of the V3–V5 or V3–V4 hypervariable regions of the env gene were sequenced with forward and reverse primers by the Sanger method. To ensure the accuracy of the results, the nucleotide sequences obtained were considered acceptable for performing subsequent analysis only if they had a chromatogram with normal raw signal intensity and no noise or abnormal peaks. For each FIV sequenced, the obtained forward and reverse nucleotide sequences were aligned with each other using the ClustalW method implemented in the BioEdit 7.2.5 software and assembled, producing a consensus sequence. The assembled nucleotide sequences were aligned using the ClustalW method with 54 GenBank reference sequences belonging to the currently recognized FIV subtypes (https://www.ncbi.nlm.nih.gov/genbank, accessed 1 December 2020, the FIV nucleotide sequences used are reported in Table S3) and translated into amino acid sequences. The assembled nucleotide sequences were also analyzed using the BLAST web interface (https://blast.ncbi.nlm.nih.gov/Blast.cgi, accessed 27 April 2022) to identify the most similar reference sequences in the GenBank database. Nucleotide diversity was calculated using DnaSP package version 5.10.01 [13] over the entire length of the nucleotide alignment, excluding sites with gaps, and compared between FIV sequences generated in this study from cats belonging to SC and AC groups, respectively. Nucleotide diversity was expressed as number of nucleotide differences per site with standard deviation (SD). The analysis was carried out on V3–V4 viral sequences only (the FIV nucleotide sequences used are reported in Table S3), because they were available for more viruses identified in both groups of cats. Potential recombination events, cysteine residues and potential N-linked glycosylation sites were detected on the V3–V5 alignment constructed with FIV sequences generated in this study and reference sequences representative for the different subtypes (the FIV nucleotide sequences used are reported in Table S3). Potential recombination events were detected using the Recombinant Detection Program (RDP) version 4.101 [14] and the SplitsTree4 program [15]. Potential N-linked glycosylation sites were predicted using the N-GlycoSite online tool (Los Alamos National Laboratories server, https://www.hiv.lanl.gov/content/index, accessed 10 March 2023) [16]. Phylogeny was carried out on V3–V5 and V3–V4 nucleotide sequence alignments (the FIV nucleotide sequences used are reported in Table S3) using MEGA version 11.0.10 [17]. Phylogenetic trees were constructed using the Neighbor-Joining method and the Tamura 3-parameters model with gamma distribution. The robustness of individual nodes was estimated using 1000 bootstrap replicates.

### 2.4. Statistical Analysis

The data were evaluated using standard descriptive statistics and reported as median and range. Categorical data were analyzed using the Chi-squared test. Continuous data (age) were assessed for normality both graphically and using the D’Agostino–Pearson test, and nonparametric statistics (Mann–Whitney U test) were used to compare groups. Statistical significance was set at *p* < 0.05. Statistical analysis was carried out using a commercially available software package (MedCalc Statistical Software version 16.8.4, MedCalc Software bvba, Ostend, Belgium).

## 3. Results

### 3.1. Study Population

Fifty cats that tested positive for the presence of anti-FIV antibodies between 2018 and 2021 in a VTH in northern Italy were included in the study. Signalment data, clinical signs, clinicopathological findings and feline leukemia virus (FeLV) antigen test results are reported in expanded and aggregated form in Table 2 and Table 3, respectively.

The enrolled subjects were mostly domestic short-hair male cats, with a median age of 8 years and 10 months (range < 1–20 years). Three of 50 (6%) cats tested positive for FeLV antigens. Thirty-three of 50 (66%) cats were grouped in the SC group and 17/50 (34%) were grouped in the AC group. No statistical association was found regarding the presence of clinical signs or clinicopathological abnormalities referable to FIV infection and signalment data, except for the age, which was significantly higher for symptomatic cats (*p* < 0.0001, Table 3).

### 3.2. Detection of FIV Proviral DNA

Detection of proviral FIV DNA in whole blood samples of seropositive cats was attempted by using six end-point PCR (PCR) assays amplifying a highly conserved fragment of the FIV genome (LTR region, one assay) or a highly variable fragment of the FIV env gene (five assays, named from A to E). All 50 cats showed at least one positive result in one of the six PCR assays. Table 4 reports the results obtained from the different end-point PCR assays.

In particular, 46/50 (92%) cats tested positive for FIV DNA by the LTR region PCR assay, which proved to be the most sensitive test used, and 43/50 (86%) tested positive for FIV DNA in at least one of the five env gene PCR assays. Thirty-nine of 50 (78%) cats tested positive by both LTR region and env gene PCR assays, 7/50 (14%) tested positive in the LTR region PCR assay only, and 4/50 (8%) tested positive in env gene PCR assays only (lab ID: 303/2019, 1127/2019, 1142/2019 and 1143/2019). As reported in Table 4, the two env gene PCR assays adopting partially modified primers to increase their specificity towards FIV subtype B (named B and D) showed better performance than the corresponding ones with unmodified primers (named A and C). This result reflects an improvement in the diagnostic performance of env PCR assays targeting subtype B in the epidemiological scenario that characterizes the geographical area assessed in this study.

### 3.3. Sequence Analysis

Nucleotide sequences of the V3–V5 hypervariable regions of the FIV env gene were obtained from 12/50 cats (lab ID: 314/2018, 402/2018, 405/2018, 304/2019, 308/2019, 309/2019, 310/2019, 1138/2019, 1140/2019, 1148/2020, 1150/2020 and 137/2021; GenBank ID: OP546000-OP546011), and they were of about 650 nucleotides (nts) in length. Eleven of these sequences were obtained from symptomatic cats, and one was from an asymptomatic cat. For another 17/50 cats, only partial nucleotide sequences, corresponding to V3–V4 hypervariable regions of the FIV env gene, were obtained (lab ID: 396/2018, 398/2018, 403/2018, 406/2018, 409/2018, 306/2019, 311/2019, 312/2019, 323/2019, 1136/2019, 1142/2019, 1143/2019, 1144/2020, 1146/2020, 1151/2020, 1152/2020 and 1197/2020; GenBank ID: OP546012-OP546028), and they were of about 450 nts in length. Ten of these sequences were obtained from symptomatic cats, and seven were from asymptomatic cats.

Of the four cats that tested negative in the LTR region PCR assay and positive in the env gene PCR assays, specific FIV env sequences were obtained for two cats (1142/2019 and 1143/2019), whereas, for the other two cats (303/2019 and 1127/2019), the nucleotide sequences obtained were not specific for the FIV genome. Therefore, FIV proviral DNA was overall detected in 48/50 (96%) of the seropositive cats tested.

Comparison of nucleotide diversity between FIVs identified in cats belonging to SC and AC groups evidenced comparable values, with 9.8 × 10^−2^ differences per site (SD 1.3 × 10^−2^) for viruses identified in symptomatic cats and 8.8 × 10^−2^ differences per site (SD 1.1 × 10^−2^) for viruses identified in asymptomatic cats.

RDP and SplitsTree analyses predicted a potential recombination event only for FIV detected in cat 308/2019 (Figure S1). Furthermore, the SplitsTree showed the lack of close relationships between the FIV detected in cat 405/2018 and all the currently known viral subtypes and its apparent correlation with the TR-Mi strain identified in Turkey in 2009 (HM639739). All the viral sequences obtained in this study showed the same cysteine residues in the hypervariable regions V3–V5 of the analyzed reference viruses, and the pattern of potential N-linked glycosylation sites was mostly comparable to that of the analyzed reference viruses (Table S4). Nevertheless, the viruses identified in three cats had a different positioning of some predicted N-linked glycosylation sites, specifically in position 200 in 405/2018, 132 and 196 in 308/2019, and 197 and 202 in 1140/2019. From these results, different and distinctive genetic characteristics emerged for these three viruses compared to all the others identified in this study.

**Figure 1:**
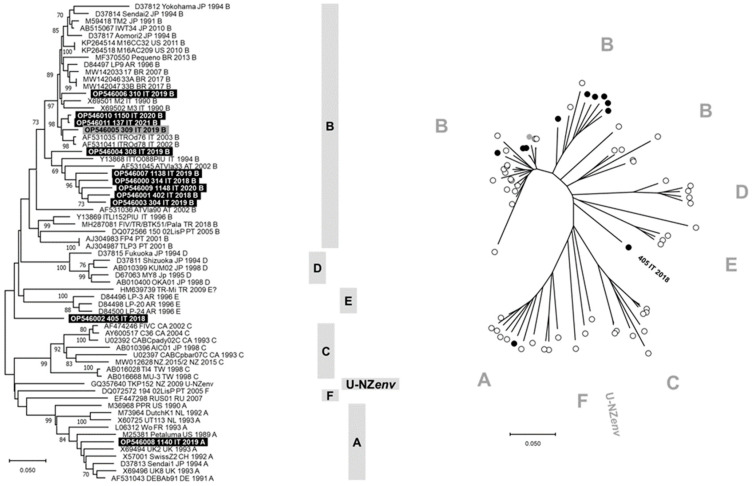
Phylogenetic tree based on the nucleotide sequences of V3–V5 hypervariable regions of env gene of feline immunodeficiency virus (FIV). Bootstrap values ≥ 70% are indicated on the respective branches. The scale bars indicate the estimated numbers of nucleotide substitutions. On the left, a traditional rectangular branch style of the tree. Identification of the sequences uses the following nomenclature: GenBank accession number, strain, country (AR: Argentina, AT: Austria, AU: Australia, BR: Brazil, CA: Canada, CH: Switzerland, CN: China, DE: Germany, FR: France, IT: Italy, JP: Japan, KR: South Korea, NL: The Netherlands, NZ: New Zeeland, PT: Portugal, RU: Russia, TR: Turkey, TW: Taiwan, UK: United Kingdom, US: United States of America), collection date (or date of database submission), and subtype. On the right, a radiation branch style of the tree. The FIV subtypes are reported as gray bars or letters. Highlighted in black or black circles: sequences generated in “symptomatic cats” (SC group) in this study. Highlighted in gray or gray circles: sequences generated in “asymptomatic cats” (AC group) in this study.

The phylogenetic tree constructed with V3–V5 nucleotide sequences allowed us to identify seven clusters consistent with genetic subtypes from A to F and U-NZenv (Figure 1). Ten of 12 FIV sequences generated in this study grouped with subtype B viruses from different countries, including the FIV identified from cat 308/2019 that formed a separate branch within this cluster. Differently, the FIV identified from cat 1140/2019 grouped in the subtype A cluster and the FIV identified from cat 405/2018 constituted a separate lineage, phylogenetically distant from the FIV subtypes recognized to date. The FIV sequence obtained from cat 405/2018 was phylogenetically distant from the TR-Mi strain (HM639739), and BLAST analysis of this viral sequence identified FIV reference sequences with nucleotide identity ≤87%. In Figure S2, the phylogenetic tree constructed with the V3–V4 nucleotide sequences generated in this study is reported and grouped in the subtype B cluster. Phylogeny suggests that FIV 405/2018 could belong to a new viral subtype. The identification of multiple subtypes changes the current knowledge on the epidemiology of FIV in northern Italy.

## 4. Discussion

In this study, blood samples of 50 FIV seropositive cats were tested by molecular assays to detect the proviral DNA and genetically characterize the viruses identified. Signalment and history of the cats included in the study reflect the risk categories reported in the literature [18]: 80% of cats were male, and 98% of cats were over 2 years old, with a median age of 8 years and 10 months. The majority (66%) of cats had clinical signs or clinicopathological abnormalities potentially referable to FIV infection, and age was significantly higher in the symptomatic cats group than in the asymptomatic cats group. This finding could be justified by the development of clinical disease later in cat life [4]. The presence of proviral DNA was confirmed by PCR in blood samples of 48/50 (96%) seropositive cats tested.

The env gene PCR assays adopted in this study allowed the detection of FIV proviral DNA, with specific amplicon sequences, in two cats that tested negative by the LTR region PCR assay. The other two cats that tested negative by the LTR region PCR assay were positive by the env gene PCR assays, but the generated nucleotide sequences were not specific for FIV. These results suggest, at first instance, that the rapid ELISA test for anti-FIV antibody detection may have produced false positive results in two cats, or that these samples may have had low amounts of DNA due to time and storage degradation. To clarify the infection status of these cats, it would have been useful to carry out a Western blot analysis [18], but this was not possible due to a lack of blood samples. The results obtained suggest that a molecular assay targeting a single FIV gene does not allow the detection of the proviral DNA in all cats, but that assays targeting different viral genome regions should be used to rule out false negative results. Furthermore, the combination of molecular assays with sequencing should be used to rule out false positive results. These difficulties linked to the high genomic variability of FIV highlighted the need for comprehensive diagnostic approaches [19,20]. This integrated approach is crucial to effectively monitoring the FIV epidemiological situation, safeguarding feline health and reducing the risk of viral spread among the feline population [21].

The majority of the viruses sequenced in this study (27/29) belonged to the FIV subtype B, corroborating previous Italian epidemiological data [8,22]. Differently, the FIV identified from a cat in 2019 (1140/2019) grouped phylogenetically with subtype A viruses. To the best of our knowledge, subtype A has never been reported in Italy [23]. Studies regarding the Italian epidemiological situation related to the prevalence of the FIV infection do not to characterize the circulating subtypes [24]. This information is essential to have appropriate diagnostic methods and to develop a vaccine. The FIV identified from a cat in 2018 (405/2018) differed from all currently known subtypes; the low percentage of identity suggests that it could belong to an unreported viral subtype. Also, SplitsTree analysis suggested that FIV 405/2018 could belong to a subtype never previously reported, potentially related to a strain identified in Turkey in 2009 (TR-Mi, HM639739) but not classified with certainty in the recognized viral subtypes [25]. The identification of an additional FIV subtype emphasizes the FIV diversity and the plasticity of its genome in generating new variants. Moreover, recombination between different subtypes may develop in areas where more than one subtype is present with the potential to create new transmittable variants with novel pathogenic properties. Since the fragment of the viral genome analyzed in this study was relatively short (hypervariable V3–V5 regions of the env gene), this potential new FIV subtype needs to be confirmed. Intensified screening of the cat population could allow the detection of other genetically related viruses and sequencing of larger regions of the FIV genome. The accuracy of diagnostic assays based on viral nucleic acid may be affected by variability in the target sequence, and therefore an understanding of subtype variation is required [26]. In particular, the presence of multiple FIV subtypes in northern Italy raises questions regarding the accuracy of the diagnostic tests currently used in this geographical area. A careful evaluation of their performance should be guaranteed to avoid the underestimation of infection cases and the ineffectiveness of disease prevention and control plans.

Sequence variability, calculated on the V3–V4 hypervariable regions of the env gene, was comparable in viruses identified in symptomatic and asymptomatic cats. This result suggests equal selective pressure on viruses infecting the two groups of cats. This result may have been influenced by the classification of cats into symptomatic and asymptomatic. Indeed, cats with clinical signs compatible with FIV infection but not caused by it could have been included in the SC group, and cats with clinical signs, such as trauma, masking underlying diseases compatible with FIV infection could have been included in the AC group. Predictive analyses revealed that an FIV belonging to subtype B identified in a cat in 2019 (308/2019) might be of recombinant origin, a frequent evolutionary event responsible for the high genetic variability that characterizes these viruses [7,27,28]. Interestingly, this cat was co-infected with FeLV; this condition has been already reported, but the consequences of co-infection have never been investigated [29,30,31]. FIV 308/2019 could be an intra-subtype recombinant, as its env nucleotide sequence is phylogenetically assigned to subtype B but in a separate clade from the other FIVs belonging to subtype B that were sequenced in this study. The FIVs obtained in the present study showed perfect conservation of the cysteine residues in the V3–V5 hypervariable regions [8]. Differently, the viruses identified in cats 405/2018, 308/2019 and 1140/2019 showed a different positioning of some predicted N-linked glycosylation sites compared to the other FIVs belonging to subtype B that were sequenced in this study. Glycosylation sites play an important role in viral infectivity and antibody-mediated neutralization [8,32,33], and variations in the glycosylation pattern may be consequent to the evolutionary process or from viruses belonging to different subtypes.

A limitation of the present study was the geographical distribution of the enrolled cats, mostly from the Emilia-Romagna region, which was linked to the VTH location. Consequently, the cat population analyzed does not adequately represent all of northern Italy but allowed us to outline the epidemiological situation of the region to which the VTH refers and update the Italian situation regarding current circulating subtypes.

## 5. Conclusions

This study confirms the need for an integrated approach that adopts different molecular tests to accurately detect the proviral FIV DNA in seropositive cats infected by genetically distinguishable viruses. This comprehensive diagnostic approach is essential to properly monitor the epidemiological situation of FIV, maintain feline health, and mitigate the risk of viral dissemination within the feline population. Furthermore, different FIV subtypes circulating in northern Italy were revealed, mainly the subtype B but also the subtype A, the latter never previously reported. A new taxon, which may represent an additional FIV subtype, was also identified. Acquiring information regarding the epidemiology of the territory and the viral evolution of circulating subtypes is essential for the development and use of adequate diagnostic and control methods.

## Figures and Tables

**Table 1:** Primers used for amplification and sequencing of FIV LTR region and env gene.

PCR and Primers	Nucleotide Sequence (5′-3′)	Genome Position ^a^	Fragment Size (Base Pairs) ^a^
LTR region PCR			
FIV_LTR_F	AGCTGCYTAACCGCRAAACCACATC	116–140	259
FIV_LTR_R	TCCCTGTTCGGGCGCCAACTG	354–374	
env gene A (V3-V4) PCR			
FIV_env_SU3	ATWCCAAAATGTGGATGGTGG	7316–7336	493
FIV_env_SU4	AATAAGGTCATCTACCTTCAT	7787–7807	
env gene B (V3-V4) PCR			
FIV_env_SU3	ATWCCAAAATGTGGATGGTGG	7316–7336	493
FIV_env_SU4_modB	AATAAGGTC**C**TCTAT**T**TTCAT	7787–7807	
env gene C (V4-V5) PCR			
FIV_env_SU5	AATCCTGTAGATTGTACCATG	7721–7741	283
FIV_env_SU6	TCCTGCYACTGGRTTATACCA	7982–8002	
env gene D (V4-V5) PCR			
FIV_env_SU5_modB	AA**C**CC**G**GTAGATTGTAC**T**ATG	7721–7741	283
FIV_env_SU6	TCCTGCYACTGGRTTATACCA	7982–8002	
env gene E (V3-V5) PCR			
FIV_env_SU3	ATWCCAAAATGTGGATGGTGG	7316–7336	687
FIV_env_SU6	TCCTGCYACTGGRTTATACCA	7982–8002	

Primers FIV_env_SU3–SU6 were published by Kann and colleagues [12]. The two primers FIV_env_SU4_modB and FIV_env_SU5_modB were partially modified to increase their specificity towards subtype B (the viral variant known to be circulating in Italy). The modified nucleotides are in bold and underlined. ^a^ Primer positions and fragment size refer to the nucleotide sequence of FIV reference strain Petaluma (GenBank ID M25381).

**Table 2:** Signalment data, clinical signs and clinicopathological findings, and year of sampling of FIV seropositive cats included in the study.

Cat	Year of Sampling	Breed	Sex	Age	Group	Clinical and Clinicopathological Findings Referable to FIV Infection	Concomitant Diseases	FeLV Antigens Test
313/2018	2018	DSH	MC	5y 11m	SC	Anemia, sarcoma of the right anterior limb		Neg
314/2018	2018	DSH	MC	17y 8m	SC	Anemia, azotemia	Hypovolemic shock	Neg
396/2018	2018	DSH	F	16y 11m	SC	Anemia, stomatitis, fever		Neg
397/2018	2018	DSH	MC	9y 6m	SC	Anemia, azotemia		Neg
398/2018	2018	DSH	M	6m	SC	Stomatitis, gastroenteritis		Neg
402/2018	2018	DSH	FS	10y 5m	SC	Dysorexia, weight loss, dermatitis		Neg
403/2018	2018	DSH	M	5y 3m	SC	Anorexia, lymphadenomegaly		Neg
405/2018	2018	DSH	MC	8y 6m	SC	Lymphoma		Neg
406/2018	2018	DSH	MC	8y 11m	AC		Congestive heart failure	Neg
407/2018	2018	DSH	F	8y 1m	SC	Blindness, azotemia, neurological signs		Neg
408/2018	2018	DSH	MC	10y 2m	SC	Rhinitis	Constipation	Neg
409/2018	2018	DSH	FS	15y 10m	SC	Anorexia, depression, fever	hepatic lipidosis	Neg
303/2019	2019	DSH	FS	15y 7m	AC			Neg
304/2019	2019	DSH	MC	13y 11m	SC	Nasal adenocarcinoma		Neg
305/2019	2019	DSH	MC	16y 4m	SC	Anemia	Dysuria	Neg
306/2019	2019	DSH	M	4y 4m	AC		Trauma	Neg
307/2019	2019	DSH	FS	17y 11m	SC	Itching, dermatitis		Neg
308/2019	2019	DSH	MC	9y 2m	SC	Anorexia, stomatitis, rhinitis		Pos
309/2019	2019	DSH	M	3y 7m	AC		Trauma	Neg
310/2019	2019	DSH	MC	11y 10m	SC	Anorexia, vomiting, fever		Neg
311/2019	2019	DSH	MC	12y	SC	Stomatitis		Neg
312/2019	2019	DSH	M	6y	AC		Trauma	Neg
318/2019	2019	DSH	MC	5y 3m	AC		Urinary tract obstruction	Neg
323/2019	2019	DSH	M	3y 7m	AC		Trauma	Pos
1127/2019	2019	DSH	M	15y 11m	AC		Trauma, septic shock	Pos
1136/2019	2019	DSH	M	10y 5m	SC	Stomatitis with ulcerations, AKI/CKD		Neg
1137/2019	2019	DSH	MC	20y 1m	SC	Dysorexia, stomatitis	Hydronephrosis, ureteral obstruction	Neg
1138/2019	2019	DSH	FS	11y 9m	SC	Fever, depression, stomatitis, sublingual neoformation		Neg
1140/2019	2019	DSH	MC	8y 10m	SC	Anemia, azotemia		Neg
1142/2019	2019	DSH	MC	3y 4m	AC		Hypertrophic cardiomyopathy	Neg
1143/2019	2019	DSH	MC	13y 11m	SC	Fever, oral ulcerations, depression	Hypertrophic cardiomyopathy	Neg
399/2020	2020	DSH	MC	7y 10m	AC			Neg
400/2020	2020	DSH	MC	16y 6m	SC	Dysorexia, weight loss, mast cell tumor		Neg
401/2020	2020	DSH	M	10y 11m	SC	Fibrosarcoma		Neg
404/2020	2020	DSH	MC	2y 11m	AC			Neg
1144/2020	2020	DSH	MC	10y 1m	AC		Trauma	Neg
1145/2020	2020	DSH	MC	14y 1m	AC		Trauma	Neg
1146/2020	2020	DSH	FS	6y 4m	SC	Anemia, depression, anorexia		Neg
1147/2020	2020	DSH	M	4y	AC		Trauma	Neg
1148/2020	2020	DSH	MC	6y 5m	SC	Anemia, stomatitis		Neg
1149/2020	2020	DSH	FS	3y 6m	SC	Lymphadenomegaly, severe dermatopathy	Cardiogenic pulmonary edema	Neg
1150/2020	2020	DSH	MC	3y 1m	SC	Stomatitis with oral ulcerations		Neg
1151/2020	2020	DSH	MC	5y 4m	SC	Dermatopathy, lymphadenomegaly		Neg
1152/2020	2020	DSH	MC	10y 8m	SC	Stomatitis, azotemia, proteinuria	Constipation	Neg
1197/2020	2020	DSH	MC	7y 4m	AC		Urinary tract obstruction	Neg
1204/2020	2020	DSH	MC	5y 10m	AC		Bladder lithiasis	Neg
1210/2020	2020	Persian	FS	5y 11m	SC	Thrombocytopenia		Neg
1211/2020	2020	DSH	MC	7y 7m	SC	AKI/CKD		Neg
137/2021	2021	DSH	M	2y 1m	SC	Anemia, fever		Neg
395/2021	2021	DSH	MC	10y	AC		Trauma	Neg

AC: Asymptomatic cats group. AKI/CKD: Acute kidney injury on chronic kidney disease. DSH: Domestic short-hair cat. F: Female. FS: Sterilized female. M: Male. MC: Castrated male. Neg: Negative. Pos: Positive. SC: Symptomatic cats group. y: Years. m: Months.

**Table 3:** Descriptive statistics and grouping of FIV seropositive cats included in this study.

Variables	Total	SC	AC	*p* Value
Number of cats	50	33	17	
Year of sampling				
2018	12 (24%)	11 (33.3%)	1 (5.9%)	0.1953
2019	19 (38%)	11 (33.3%)	8 (47.1%)	
2020	17 (34%)	10 (30.3%)	7 (41.2%)	
2021	2 (4%)	1 (3%)	1 (5.9%)	
Sex				
Male	40 (80%)	24 (72.7%)	16 (94.1%)	0.1562
Female	10 (20%)	9 (27.3%)	1 (5.9%)	
Age ^a^	8y 10m [6m–20y 1m]	10y 2m [6m–20y 1m]	6y [2y 11m–15y 11m]	<0.0001
Breed				
DSH	49 (98%)	32 (97%)	17 (100%)	0.7330
Persian	1 (2%)	1 (3%)	0 (0%)	
FeLV antigen test				
Positive	3 (6%)	1 (3%)	2 (11.8%)	0.5462
Negative	47 (94%)	32 (97%)	15 (88.2%)	

The chi-squared test and the Mann–Whitney U test (age) were carried out on the symptomatic and asymptomatic cats. Data are reported as n (%). ^a^ Data are reported as median [range]. Values in bold indicate statistical significance. AC: Asymptomatic cats group. DSH: Domestic short-hair cat. m: Months. SC: Symptomatic cats group. y: Years.

**Table 4:** Results obtained by the six end-point PCR assays used.

		Total (N 50)		
		Positive	Negative	
LTR region PCR		46 (92%)	4 (8%)	
env gene PCR assays		43 (86%)	7 (86%)	
env gene A (V3-V4) PCR		1 (2%)	49 (98%)	
env gene B (V3-V4) PCR		35 (70%)	15 (30%)	
env gene C (V4-V5) PCR		2 (4%)	48 (96%)	
env gene D (V4-V5) PCR		15 (30%)	35 (70%)	
env gene E (V3-V5) PCR		16 (32%)	34 (68%)	
		LTR region PCR		
		Positive	Negative	Total
env gene PCR assays	Positive	39 (78%)	4 (8%)	43 (86%)
	Negative	7 (14%)	0 (0%)	7 (14%)
	Total	46 (92%)	4 (8%)	50 (100%)

## Data Availability

All data generated or analyzed during this study are included in this published article and its supplementary information files. The nucleotide sequences generated and analyzed during the current study are available in the International Nucleotide Sequence Database Collaboration repository (INSDC, http://www.insdc.org/, accessed 30 March 2023) with the IDs: OP546000-OP546028.

## Supplementary Materials

The following supporting information can be downloaded at: https://www.mdpi.com/article/10.3390/pathogens13060463/s1, Table S1: Clinical signs and clinicopathological abnormalities potentially referable to FIV infection. Table S2: Reference nucleotide sequences of FIV available in the GenBank database used for primer design. Table S3: Reference nucleotide sequences of FIV available in the GenBank database used for sequence analysis; Table S4: Potential N-linked glycosylation sites predicted using the N-GlycoSite software; Figure S1: Potential recombination events predicted using the Recombinant Detection Program and the SplitsTree4 program, respectively; Figure S2: Phylogenetic tree on the nucleotide sequences of V3–V4 hypervariable regions of env gene of FIV.
