# Supplementary material for: Molecular Detection and Genetic Characterization of Feline Immunodeficiency Virus (FIV) in Seropositive Cats in Northern Italy
Source: Pathogens. 2024 May 31;13(6):463. doi: 10.3390/pathogens13060463 (PMC11206283; doi:10.3390/pathogens13060463)
Supplement: Supplementary file 1 [file pathogens-13-00463-s001.zip › 20240527 pathogens-2997377-supplementary.pdf]

Supplementary Materials

Molecular Detection and Genetic Characterization of Feline Immunodeficiency Virus (FIV) in Seropositive Cats in Northern Italy

Andrea Balboni [1], Veronica Facile [1], Laura Gallina [1,*], Maria Chiara Sabetti [2], Francesco Dondi [1] and Mara Battilani [1]

[1] Department of Veterinary Medical Sciences, Alma Mater Studiorum-University of Bologna, Ozzano Emilia, 40064 Bologna, Italy; a.balboni@unibo.it (A.B.); veronica.facile2@unibo.it (V.F.); f.dondi@unibo.it (F.D.); mara.battilani@unibo.it (M.B.)

[2] Department of Veterinary Sciences, University of Parma, 43126 Parma, Italy; mariachiara.sabetti@unipr.it

* Correspondence: laura.gallina@unibo.it

1. Supplementary Tables

Table S1. Clinical signs and clinicopathological abnormalities potentially referable to FIV infection.

Anorexia, depression, fever and generalized lymphadenopathy
Gingivostomatitis
Hematological abnormalities (anemia, leukopenia, neutropenia, lymphopenia and thrombocytopenia)
Hypergammaglobulinemia and proteinuria
Chronic inflammatory states of the respiratory, digestive, skin and urinary systems
Ocular disorders (conjunctivitis and uveitis)
Neurologic signs
Neoplasia
Chronic kidney disease
Reproductive disorders

Table S2. Reference nucleotide sequences of FIV available in the GenBank database used for primers design.

GenBank ID	Strain	Host	Year [a]	Geographic origin	Subtype
U11820	USIL2489_7B	cat	1995	USA	B
M25381	Petaluma	cat	1989	USA	A
M36968	PPR	cat	1990	USA	A
AF474246	BM3070	cat	2002	Canada	C
AY600517	FIV-C36	cat	2004	USA	C
MF352016	CHN17	cat	2017	China	A
MF370550	Pequeno	cat	2013	Brazil	B
NC_001482	Petaluma clone FIV-14	cat	1989	USA	A

GenBank database (https://www.ncbi.nlm.nih.gov/genbank/, accessed 01 December 2020).

[a] year of virus identification or sequence submission in the GenBank database

Table S3. Reference nucleotide sequences of FIV available in the GenBank database used for sequence analysis.

GenBank ID	Strain	Host	Geographic origin	Year [a]	Subtype	Group	V3-V5	V3-V4	ND	RCG
D37811	Shizuoka	cat	Japan	1994	D					
D37812	Yokohama	cat	Japan	1994	B					
D37813	Sendai1	cat	Japan	1994	A					
D37814	Sendai2	cat	Japan	1994	B					
D37815	Fukuoka	cat	Japan	1994	D					
D37817	Aomori2	cat	Japan	1994	B					
D67063	MY8	cat	Japan	1995	D					
D84496	LP-3	cat	Argentina	1996	E					
D84497	LP9	cat	Argentina	1996	B					
D84498	LP-20	cat	Argentina	1996	E					
D84500	LP-24	cat	Argentina	1996	E					
L06312	Wo	cat	France	1993	A					
M25381	Petaluma	cat	USA	1989	A					
M36968	PPR_USA	cat	USA	1990	A					
M59418	TM2	cat	Japan	1991	B					
M73964	DutchK1	cat	The Nederland	1992	A					
U02392	CABCpady02C	cat	Canada	1993	C					
U02397	CABCpbar07C	cat	Canada	1993	C					
X57001	SwissZ2	cat	Switzerland	1992	A					
X60725	UT113	cat	The Nederland	1993	A					
X69494	UK2	cat	United Kingdom	1993	A					
X69496	UK8	cat	United Kingdom	1993	A					
X69501	ItalyM2	cat	Italy	1990	B					
X69502	1990ItalyM3	cat	Italy	1990	B					
Y13868	ITTO088PIU	cat	Italy	1994	B					
Y13869	ITLI152PIU	cat	Italy	1996	B					
AB010396	AIC01	cat	Japan	1998	C					
AB010399	KUM02	cat	Japan	1998	D					
AB010400	OKA01	cat	Japan	1998	D					

AB016028	TI4	cat	Taiwan	1998	C	
AB016668	MU-3	cat	Taiwan	1998	C	
AB515067	IWT34	cat	Japan	2010	B	
AF474246	FIVC	cat	Canada	2002	C	
AF531035	ITROd76	cat	Italy	2003	B	
AF531036	ATVIa90	cat	Austria	2002	B	
AF531041	ITROd78	cat	Italy	2002	B	
AF531043	DEBAb91	cat	Germany	1991	A	
AF531045	ATVIa33	cat	Austria	2002	B	
AJ304983	FP4	cat	Portugal	2001	B	
AJ304987	TLP3	cat	Portugal	2001	B	
AY600517	C36	cat	Canada	2004	C	
DQ072566	150_02LisP	cat	Portugal	2005	B	
DQ072572	194_02LisP	cat	Portugal	2005	F	
EF447298	RUS01	cat	Russia	2007	NA	
GQ357640	TKP152	cat	New Zeeland	2009	U-NZenv	
HM639739	TR-Mi	cat	Turkey	2009	E?	
KP264514	M16CC32	cat	USA	2011	B	
KP264518	M16AC209	cat	USA	2010	B	
MF370550	Pequeno	cat	Brazil	2013	B	
MH287081	FIV/TR/BTK51/Pala	cat	Turkey	2018	B	
MW012628	NZ.2015/2	cat	New Zeeland	2015	C	
MW142033	17	cat	Brazil	2007	B	
MW142046	33A	cat	Brazil	2017	B	
MW142047	33B	cat	Brazil	2017	B	
OP546000	314/2018	cat	Italy	2018	B	SC
OP546001	402/2018	cat	Italy	2018	B	SC
OP546002	405/2018	cat	Italy	2018	?	SC
OP546003	304/2019	cat	Italy	2019	B	SC
OP546004	308/2019	cat	Italy	2019	B	SC
OP546005	309/2019	cat	Italy	2019	B	AC
OP546006	310/2019	cat	Italy	2019	B	SC

OP546007	1138/2019	cat	Italy	2019	B	SC				
OP546008	1140/2019	cat	Italy	2019	A	SC				
OP546009	1148/2020	cat	Italy	2020	B	SC				
OP546010	1150/2020	cat	Italy	2020	B	SC				
OP546011	137/2021	cat	Italy	2021	B	SC				
OP546012	396/2018	cat	Italy	2018	B	SC				
OP546013	398/2018	cat	Italy	2018	B	SC				
OP546014	403/2018	cat	Italy	2018	B	SC				
OP546015	406/2018	cat	Italy	2018	B	AC				
OP546016	409/2018	cat	Italy	2018	B	SC				
OP546017	306/2019	cat	Italy	2019	B	AC				
OP546018	311/2019	cat	Italy	2019	B	SC				
OP546019	312/2019	cat	Italy	2019	B	AC				
OP546020	323/2019	cat	Italy	2019	B	AC				
OP546021	1136/2019	cat	Italy	2019	B	SC				
OP546022	1142/2019	cat	Italy	2019	B	AC				
OP546023	1143/2019	cat	Italy	2019	B	SC				
OP546024	1144/2020	cat	Italy	2020	B	AC				
OP546025	1146/2020	cat	Italy	2020	B	SC				
OP546026	1151/2020	cat	Italy	2020	B	SC				
OP546027	1152/2020	cat	Italy	2020	B	SC				
OP546028	1197/2020	cat	Italy	2020	B	AC				

GenBank database (https://www.ncbi.nlm.nih.gov/genbank/, accessed 17 August 2021).

AC: Asymptomatic cats group. NA: Not available. SC: Symptomatic cats group. V3-V5: Sequences used for V3-V5 alignment and phylogeny. V3-V4: Sequences used for V3-V4 phylogeny. ND: Sequences used for nucleotide diversity evaluation. RCG: Sequences used for the detection of potential recombination events, cysteine residues and potential N-linked glycosylation sites.

[a] year of virus identification or sequence submission in the GenBank database.

Table S4. Potential N-linked glycosylation sites predicted using the N-GlycoSite software.

Position in alignment	15	61	65	91	112	124	132	142	161	174	191	194	196	197	200	202	Glycosylation Count
M25381_Petaluma_US_1989_A		N	N	N	N			N		N	N	N					**8**
D37814_Sendai2_JP_1994_B	N	N	N	N	N	N		N	N	N	N	N					**11**
AF474246_FIVC_CA_2002_C		N	N	N	N	N		N	N	N	N		N				**10**
D37811_Shizuoka_JP_1994_D		N	N	N	N			N	N	N	N	N					**9**
D84498_LP-20_AR_1996_E	N	N	N	N	N	N		N	N	N	N	N					**11**
HM639739_TR-Mi_TR_2009_E		N	N	N	N	N		N	N	N	N	N					**10**
DQ072572_194_02LisP_PT_2005_F		N	N	N	N			N	N	N	–	–	–	–	–	–	**7**
GQ357640_TKP152_NZ_2009_U-NZenv		N	N	N	N	N		N	N	N	N	–	–	–	–	–	**9**
314_IT_2018_B		N	N	N	N	N		N	N	N	N	N					**10**
402_IT_2018_B	N	N	N	N	N	N		N	N	N	N	N					**11**
405_IT_2018_		N	N	N	N			N	N	N	N	N			N		**10**
304_IT_2019_B	N	N	N	N	N	N		N	N	N	N	N					**11**
308_IT_2019_B	N	N	N	N	N		N	N	N	N	N		N				**11**
309_IT_2019_B	N	N	N	N	N	N		N	N	N	N	N					**11**
310_IT_2019_B	N	N		N	N	N		N	N	N	N	N					**10**
1138_IT_2019_B	N	N	N	N	N	N		N	N	N	N	N					**11**
1140_IT_2019_A	–	N	N	N	N	N		N	N	N	N			N		N	**11**
1148_IT_2020_B		N	N	N	N	N		N	N	N	N	N					**10**
1150_IT_2020_B	N	N	N	N	N	N		N	N	N	N						**10**
137_IT_2021_B	N	N	N	N	N	N		N	N	N	N	N					**11**
**Glycosylation Count**	**10**	**20**	**19**	**20**	**20**	**15**	**1**	**20**	**19**	**20**	**19**	**14**	**2**	**1**	**1**	**1**	**202**

The analyses were carried out on the V3-V5 alignment constructed with FIV sequences generated in this study and reference sequences representative for the different subtypes retrieved from GenBank (Table S3) used for sequence analysis by using the N-GlycoSite software available on the Los Alamos National Laboratories server (https://www.hiv.lanl.gov/content/index, accessed 01 June 2022).

N : potential N-linked glycosylation sites

– : position not included in the analysed sequence

2. Supplementary Figures

Figure S1. Potential recombination events predicted using the Recombinant Detection Program and the SplitsTree4 program, respectively.

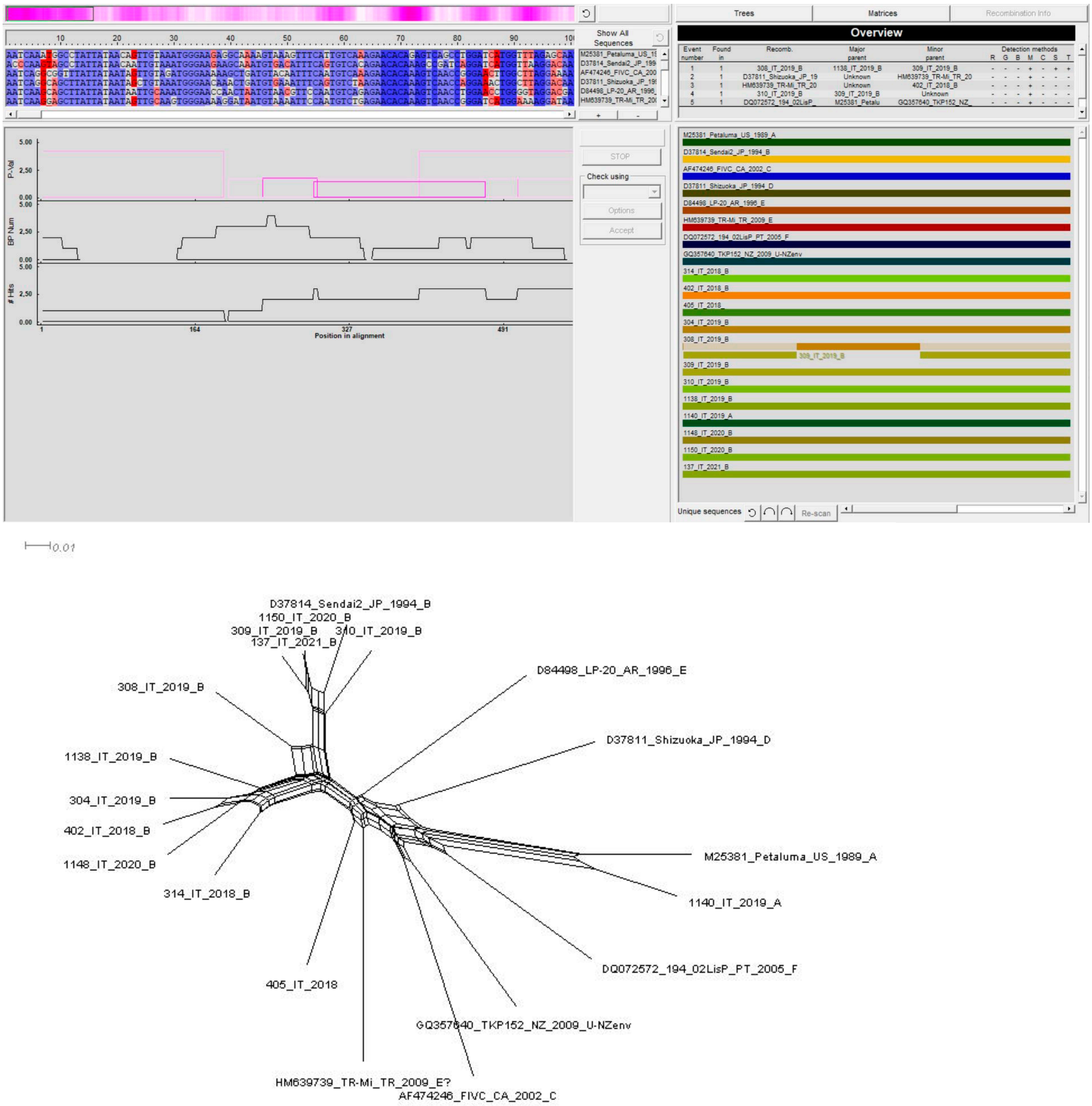

The analyses were carried out on the V3-V5 alignment constructed with FIV sequences generated in this study and reference sequences representative for the different subtypes retrieved from GenBank (Table S3), using Recombinant Detection Program (RDP) version 4.101 and the SplitsTree4 program, respectively.

Figure S2. Phylogenetic tree on the nucleotide sequences of V3-V4 hypervariable regions of env gene of FIV

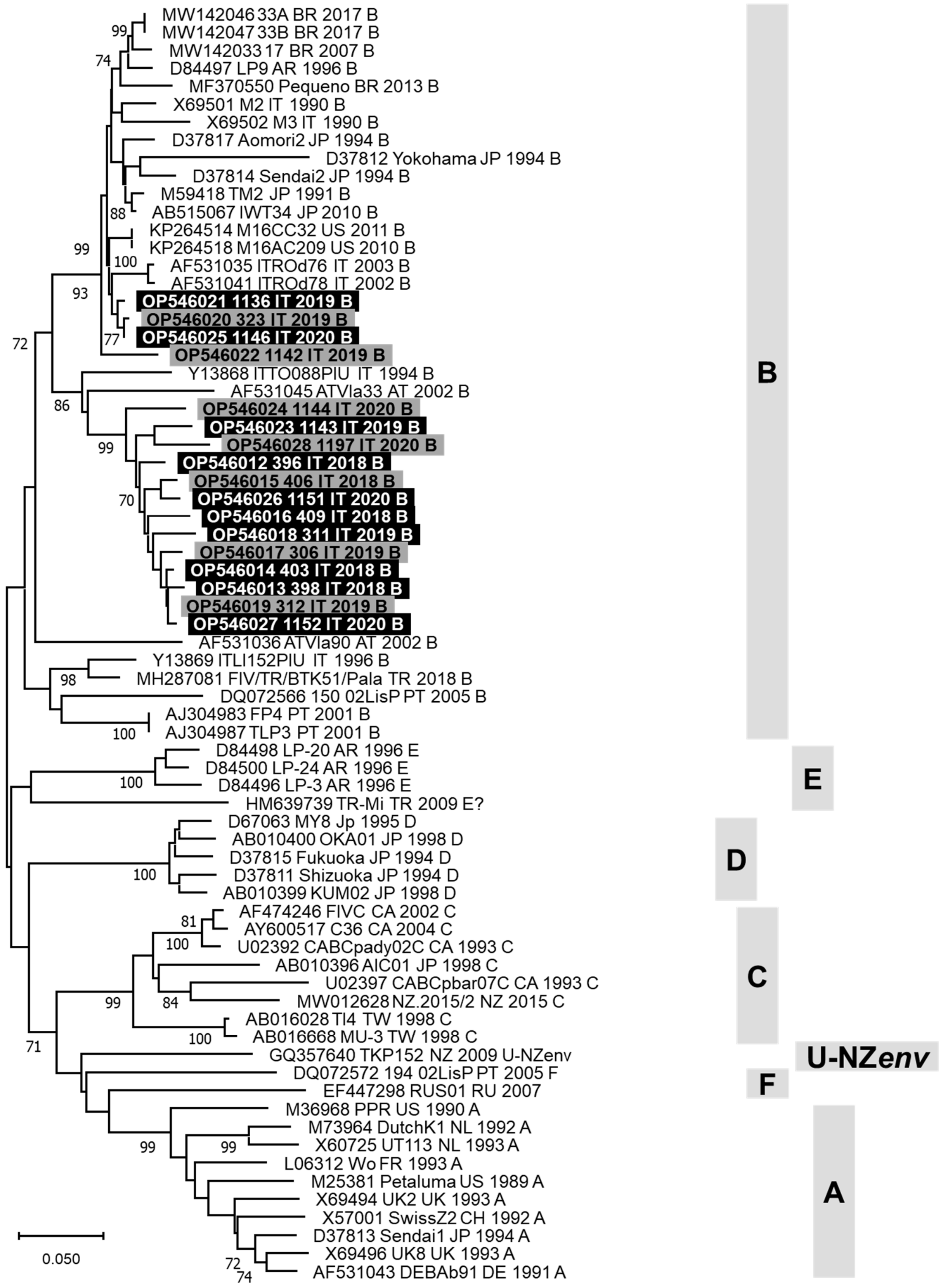

Phylogeny was carried out on FIV sequences obtained in this study and 54 reference strains (Table S3) using MEGA 11 version 11.0.10. Phylogenetic tree was constructed using Neighbor-Joining method and the Tamura 3-parameters model with gamma distribution. Statistical support was provided by bootstrapping with 1,000 replicates. Bootstrap values ⩾ 70% are indicated on the respective branches. The scale bars indicate the estimated numbers of nucleotide substitutions. Identification of the sequences undergoes the following nomenclature: GenBank accession number, strain, country (AR: Argentina, AT: Austria, AU: Australia, BR: Brazil, CA: Canada, CH: Switzerland, CN: China, DE: Germany, FR: France, IT: Italy, JP: Japan, KR: South Korea, NL: The Netherland, NZ: New Zeeland, PT: Portugal, RU: Russia, TR: Turkey, TW: Taiwan, UK: United Kingdom, US: United States of America), collection date (or date of database submission), and subtype. The FIV subtypes are reported as grey bars. Highlighted in black: sequences generated in "symptomatic cats" (SC group) in this study. Highlighted in grey: sequences generated in "asymptomatic cats" (AC group) in this study.
